# Au(100) as a Template for Pentacene Monolayer

**DOI:** 10.3390/molecules26082393

**Published:** 2021-04-20

**Authors:** Artur Trembułowicz, Agata Sabik, Miłosz Grodzicki

**Affiliations:** Department of Physics and Astronomy, Institute of Experimental Physics, University of Wrocław, 50-204 Wrocław, Poland; agata.sabik@uwr.edu.pl (A.S.); milosz.grodzicki@uwr.edu.pl (M.G.)

**Keywords:** Au(100), pentacene, coexistence of phases, metal–organic interface, STM

## Abstract

The surface of quasi-hexagonal reconstructed Au(100) is used as the template for monolayer pentacene (PEN) self-assembly. The system is characterized by means of scanning tunneling microscopy at room temperature and under an ultra-high vacuum. A new modulated pattern of molecules with long molecular axes (MA) arranged along hex stripes is found. The characteristic features of the hex reconstruction are preserved herein. The assembly with MA across the hex rows leads to an unmodulated structure, where the molecular layer does not recreate the buckled hex phase. The presence of the molecules partly lifts the reconstruction—i.e., the gold hex phase is transformed into a (1×1) phase. The arrangement of PEN on the gold (1×1) structure is the same as that of the surrounding molecular domain on the reconstructed surface. The apparent height difference between phases allows for the distinction of the state of the underlying gold surface.

## 1. Introduction

Nowadays, interfaces between organic molecules and metal surfaces are attracting a lot of attention in surface science. Metal surfaces provide the template for on-surface syntheses of novel chemical reactions/products [1,2,3,4,5,6], or self-assembly of unique molecular structures [7,8,9,10]. Moreover, the adsorption of organic compounds may affect the atomic arrangements of metal surfaces, e.g., lifting the surface reconstruction [11,12,13]. Determining the surface structure under the molecules is crucial for understanding metal–organic interactions and for indicating system reactivity. However, in the case of complicated surface atomic arrangements, such as those observed in (100) plane of gold, the determination of the structural properties of a metal–organic interface is still challenging [14,15,16].

From the topmost layer of the Au(100) surface proceeds a massive quasi-hexagonal (hex) reconstruction, the structure of which is still under investigation [17,18,19,20,21,22]. The hex-reconstructed layer has a higher packing density than the (1×1) phase; it is commonly described, in respect to the unreconstructed second layer, by simplified rectangular unit cells, such as (5×N), where N varies from 20 to 28 [17,18,22]. However, the real size of the unit cell is much bigger and determined to be c(28×48) [20]. It was shown by scanning tunneling microscope (STM) that the reconstruction exhibits long-range modulation and consists of atomic rows aligned along the <011> directions whose periodicity (along the row) is N·*a* (*a* = 2.88 Å is the surface lattice constant of the unreconstructed (100) plane of gold). The hex atoms along the row reside either on top and bridge or on hollow and bridge sites of the non-reconstructed Au(100) second layer. Therefore, the elevation and diminution of atoms within the atomic row are observed in the STM investigations, with the apparent height difference of about 40 pm, as a consequence of various atomic vertical positions. For simplified unit cells across the rows, ridges and the valleys are formed, with a periodicity of 5*a* (5·2.88 Å = 14.4 Å) above the top and hollow positions, respectively. In the hollow configuration, the surface atoms have the lowest vertical position, which is around 66 pm lower than the position of atoms located on the top. In the STM images, the hex Au(100) surface reveals a pattern composed of 5*a* wide stripes along the <011> directions. The stripes tend to be parallel to the step edges [17,18,19,20,21,22]. Apart from the massive hex reconstruction, the rotation up to 0.83° of the topmost Au(100) layer, with respect to the <011> direction, was reported [18,20,21]. The presence of rotation depends on experimental conditions, such as surface annealing during or after Ar ion sputtering. The rotation of the topmost layer is an additional parameter influencing the modulation of atomic rows [20,21]. Recently, it was shown by STM and density functional theory (DFT) investigations that ion (argon or neon) irradiation of hex Au(100) can lead to the coexistence of the hex and unreconstructed (1×1) phases on the topmost layer [22]. Both STM and DFT results show that the (1×1) phase appears about 0.6–0.9 Å lower than the hex structure. Changing the conditions of bombardment (i.e., mass of ion, ion energy or time of exposition) allows one to tune the relative proportions of the (1×1) and the hex phases on the surface.

It was shown that the adsorption of adatoms or molecules could lift the hex reconstruction of Au(100) [11,23,24,25,26]. In the case of phenylacetylene molecules, the hex reconstruction was found to be completely transformed to the (1×1) phase under the molecular domains [11]. The indication of partially lifting the hex structure was reported for the monolayer (ML) of α-sexitiophene (α-6T) [24]. Small stripe-like regions—which appear about 0.7 Å lower than the molecules in large domains—were observed by STM on surface terraces therein. The α-6T molecules in the stripe-like regions exhibit a different structure than those in the large domains. For α-6T in the gold hex phase, the variation of molecules STM contrast was observed [25]. On the other hand, in the STM investigations on the organization of molecules, such as metal-phthalocyanines (MPcs), or pentacene (PEN), or their mixture, it is usually impossible to find out if the Au(100) surface under the molecular layer is reconstructed or not [14,15,16]. For instance, 1 ML of PEN on Au(100) reveals the homogenous molecular rows. There is no visible modulation that would be assigned to the reconstruction of the underlying surface [14]. In the case of the co-adsorption of hexadecaflouro copper phthalocyanine (F_16_CuPc) and PEN with a molecular ratio of 1:1, the hex reconstructed Au(100) layer was observed by STM in the uncovered substrate regions [16]. However, under the bimolecular islands, the reconstruction was no longer visible; thus, ascertainment of the STM data from the surface phase was not possible [16]. Contrary to the Au(100), the STM images of ordered islands of PEN or MPc on the Au(111) surface, from which massive $\left(23\times \sqrt{3}\right)$ reconstruction also proceeds, usually reveal the herringbone pattern of substrate, e.g., [5,27,28,29,30,31,32].

Since the phase of Au(100) under the PEN layer is still an open question, in this work, we employed the STM to investigate the structural properties of interfaces between 1 ML of PEN and Au(100). We show that two arrangements of molecules on the reconstructed Au(100) surface could be distinguished, i.e., with the long molecular axis (MA) across and along the atomic rows of the hex structure. Only for the latter is the modulation of hex reconstruction clearly unveiled, which, according to our knowledge, was not previously observed in PEN on Au(100). Furthermore, we show the indications of partially lifting the surface reconstruction induced by the molecules.

## 2. Results and Discussion

**Figure 1:**
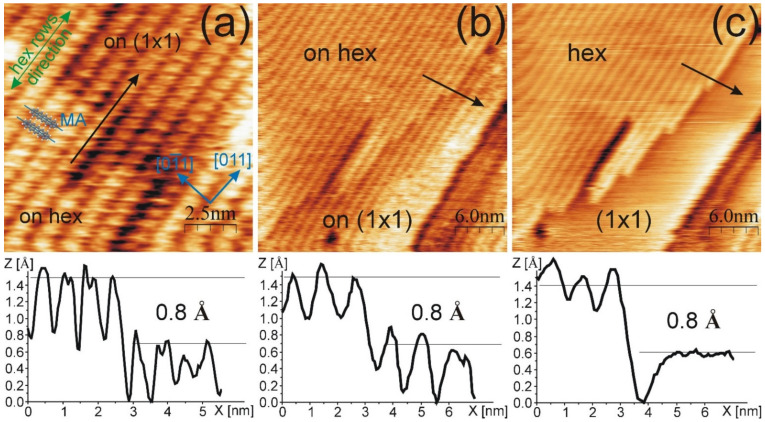
STM image of (**a**,**b**) non-modulated PEN structure; (**c**) surface area from (**b**), but with no visible PENs on the hex nor on the (1×1) phase. For each image, the height profile is drawn along the black arrow and provided below the image. Images taken for (**a**) U = 0.41 V, I = 3.85 nA; (**b**,**c**) U = −0.25 V, I = 3.85 nA.

After the deposition of 1 ML of PEN on the hex reconstructed Au(100), molecular rows are present on the surface. In the STM images in Figure 1a,b, the non-modulated structure is presented. For this structure, the molecular rows are oriented along the <011> crystallographic direction. A single row is assigned by the black arrow in Figure 1a. The side-to-side distance between molecules in the same row is 6.9 ± 0.5 Å, while the end-to-end distance between PEN in the adjacent rows is 14.7 ± 0.5 Å. The layer is well-ordered, and no modulation through or across molecular rows is observed. Thus, the determination of the Au phase under the molecular layer is not directly possible with the STM images. However, there are sample areas where the PEN rows are located about 0.8 Å lower (in a depression) than the rest of the ordered domain, as illustrated by the height profile drawn along the black arrow in Figure 1a. This value is in good agreement with the apparent height difference between the hex and (1×1) phases of bare and ion-modified Au(100) surfaces, as reported in Ref. [22]. This finding suggests that for the region labelled as “on hex” (protrusion) in Figure 1a, the hex phase of Au(100) is present underneath the molecular layer, whereas under the layer located in the depression, the unreconstructed surfaces coexist.

**Figure A1:**
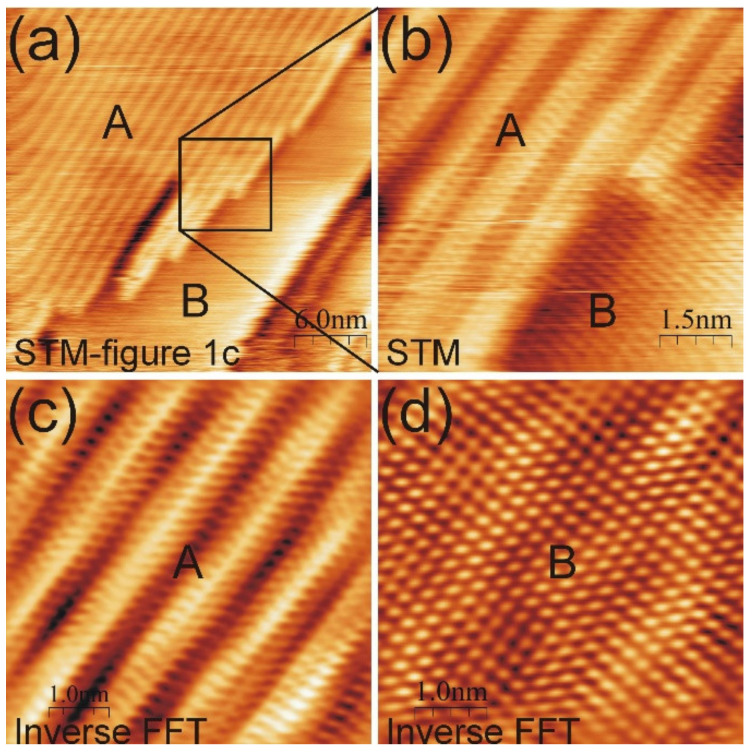
(**a**) Copy of Figure 1c from the main text; (**b**) magnification of Figure A1a indicated by the square; (**c**,**d**) inverse FFT of areas marked as A and B, showing the presence of the hex and (1 × 1) phases of Au(100), respectively.

The coexistence of the hex and (1×1) Au(100) phases under the PEN layer is confirmed by observing the continuous scanning of a given surface area at a relatively high (for molecular layers) tunneling current of 3.85 nA. The first scan is shown in Figure 1b. Similarly, as shown in Figure 1a, the PEN domains located on the protrusion and in the depression of wide terraces are visible and assigned as “on hex” and “on (1×1)”, respectively. Sequential imaging tends to reveal a bare Au(100) surface on both protrusion and depression areas (see Figure 1c). Such observation could be explained by the tip-induced removal of PEN molecules, observed previously for phthalocyanine on the TiO_2_(011) surface [33], or by the tunneling of electrons through the PEN layer, as was reported for the hexagonal boron nitride (h-BN) layer [34]. Regardless of the reason, the STM image shows the template, i.e., the coexistence of the hex and (1×1) phases, as visible in Figure 1c. Zoomed images of Figure 1c are provided in Appendix A (see Figure A1). The comparison of images in Figure 1b,c unveils that the location of the (1×1) Au phase corresponds to the PEN domains in the depression, whereas the PEN domains located on the protrusion are adsorbed on the hex-reconstructed surface. The height profiles drawn along the black arrows in the STM images in Figure 1b,c show that the apparent height difference between the hex and (1×1) phases is about 0.8 Å for PEN, on covered as well as on bare Au(100) surfaces. Uncovering the Au(100) surface also reveals that the PEN molecules are arranged with their long MA across the hex surface atomic rows, and in the width of a single stripe, one molecule is adsorbed. However, it is difficult to determine the precise angle between the MA and hex row directions from the STM appearance of PEN. The ball model for two PENs, where the MA is along the [0$\bar{1}$1] direction, is shown in Figure 1a, which illustrates the proposed PEN configuration. PEN molecules located in the (1×1) phase have the same arrangement as those observed in the hex structure. Moreover, the molecular rows in the (1×1) phase are aligned with those on the reconstructed surface. In Figure 1a, seven molecular rows are in the depression, which corresponds to the transformation of seven Au(100) stripes into the (1×1) phase.

**Figure 2:**
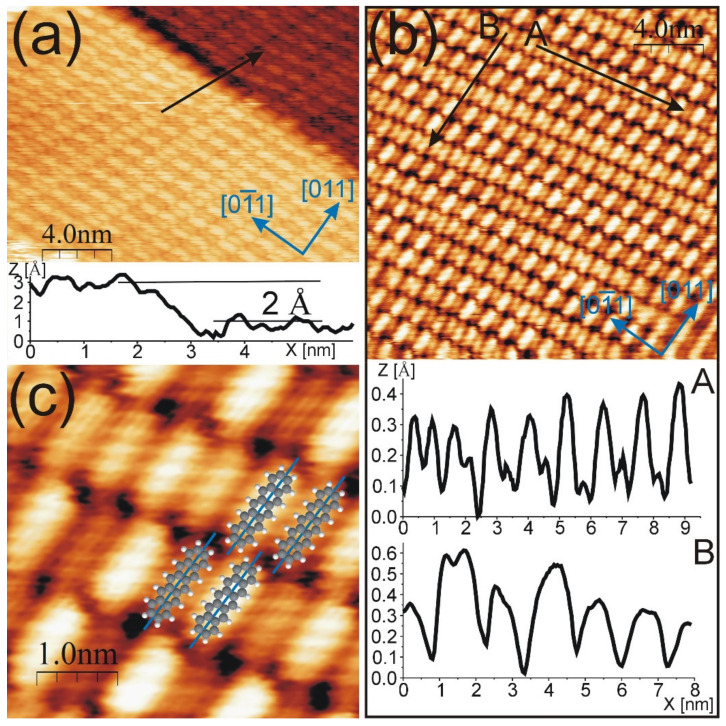
STM images of (**a**) terraces covered by modulated structure; (**b**) modulated structure—the complexity of modulation is visible. Two height profiles along the black arrows labelled as A and B are provided below the image; (**c**) modulated structure with PEN ball models inserted in bright and dark molecular features. Taken for (**a**) U = −1.35 V; I = 0.25 nA; (**b**,**c**) U = 2.00 V; I = 0.51 nA.

Apart from the non-modulated structure, surface areas covered by PEN domains with the molecular STM contrast modulation were found. The representative STM images are presented in Figure 2.

In Figure 2a, two surface terraces covered with a PEN modulated structure are visible. The height difference between upper and lower terrace is about 2.0 Å (see the height profile drawn along the black arrow). The molecules exhibit various contrasts. More precisely, the molecular rows consist of bright and dark molecular species assembled in an alternating manner. In the neighboring bright and dark molecules, the PEN ball models are as shown in Figure 2c. The STM images shown in Figure 2b,c, as well as the height profiles drawn along the black arrow in Figure 2b, clearly reveal that the contrast of molecules is not constant along and across the PEN rows. The profile drawn along the arrow labelled as A presents the variation in the apparent height difference between neighboring molecules in the same row. The first two molecules visible on the left exhibit comparable apparent heights. Moving to the right, the apparent height difference between the nearest neighbors increases; for the two molecules located on the right end of the profile, this increase is equal to 30 pm (not the maximum value). The apparent height difference between neighboring bright and dark molecular species varies from several to 50 pm. This interval is in accordance with the apparent height difference between atomic rows of bare Au(100) surfaces [21]. The distance between the nearest neighbors in the same row (i.e., side-to-side distance) is 7.3 ± 0.2 Å, which, within the experimental error, is equal to 2.5*a* (i.e., 7.2 Å) and is equivalent to half of the width of the hex stripe. By tracking the black solid line labelled as B in Figure 2b, it can be observed that between the neighboring rows, the molecules are not aligned. The end-to-end distance is 15.2 ± 0.3 Å. For the modulated structure, the angle between the direction of molecular rows and the <011> surface crystallographic directions is not constant. For the arrangement visible in Figure 2a, this angle is equal to 25 ± 3°, whereas for the structure shown in Figure 2b, it is 18 ± 3°. It was shown in Ref. [20] that small angles of rotation of the hex layer have a significant influence on the Au(100) pattern. The 0.83° rotation of the hex layer gives rise to the inclination of the corresponding maxima (or minima) of atomic rows by 22° [20]. The range of rotation of PEN rows is in agreement with the inclination angles of the Au(100) pattern. The direction of long MA is close to the <011> direction. Moreover, in Figure 2a, it can be observed that the MA is arranged along the step edge of the surface terrace, which tends to be parallel to the hex stripes. The presence of modulation indicates that there is a reconstructed Au(100) surface under the molecular layer. From the above described similarities between PEN and bare Au(100) patterns—namely, apparent heights, row and stripe directions, and side-to-side molecule distance—we conclude that in this structure, PENs are adsorbed along the atomic rows of the Au(100) surface. In such a configuration, one of the contributions to the molecular modulation is the PEN location with respect to the atomic rows, i.e., on ridges and in valleys of the hex-Au(100). The second factor affecting the STM appearance is the electronic structure of the PEN–Au interface. To determine whether the electronic properties have a significant influence, additional experimental and theoretical investigations are necessary. However, there are some similarities in the modulation observed in the PEN-modulated structure and the bare hex-Au(100). The length of the height profile drawn along the <011> direction (the black arrow labelled as B) in Figure 2b is about 80 Å and is comparable to the length of a simplified hex-Au(100) unit cell (28 × 2.88 Å ≈ 81 Å). The profile (arrow) goes through three flattened areas, i.e., the areas where the neighboring molecules (in the same row) have comparable heights. The flattened areas are at the beginning, in the middle, and at the end of the profile. Only for the outers is the arrow located between molecules. Therefore, the periodicity of PENs along the <011> direction is comparable to that observed in the bare hex-Au(100). Moreover, the hex structure of Au(100) consists of the local topographic flattening of every half of the simplified unit cell length [20], such as that found within the PEN structure. The comparison of PEN modulation—detected within the single molecular row—to the bare surface requires taking into account the complex long-range modulation of the hex-Au(100) reconstruction, which is not included in the simplified (5×N) unit cell. In Ref. [20], it was shown that simplified (5×N) unit cells are unsuited for describing the hex-Au(100) superstructure. This gives an additional contribution to the variation of molecular contrast within the same row. Similarly for PEN, the alternately brighter and darker STM appearance due to Au(100) buckling was previously reported for 1 ML of α-6T [25].

**Figure 3:**
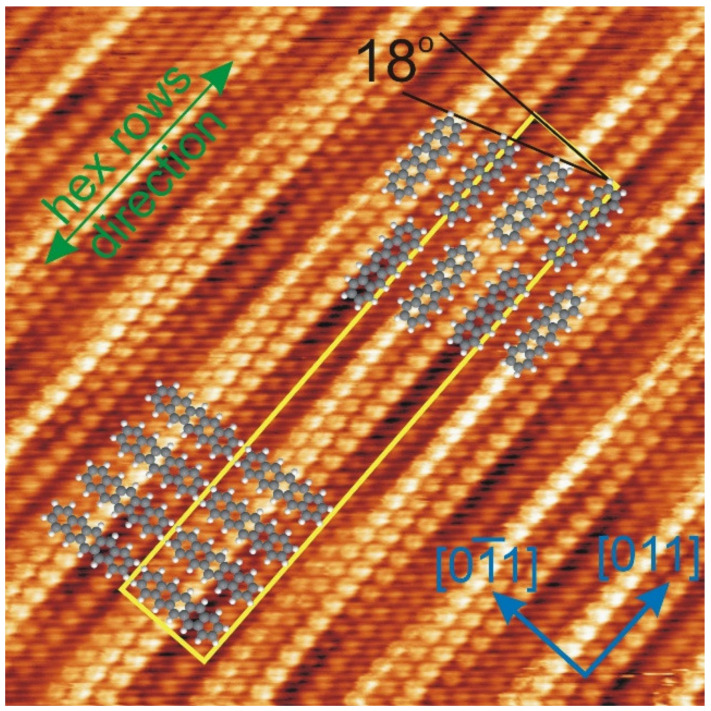
A 7 × 7 nm^2^ STM image of Au(100) with the atomic resolution. The PEN ball models arranged along and across the stripes were inserted. Taken for U = −0.02 V, I = 6.54 nA.

The modulated structure is the second PEN arrangement on the hex-reconstructed Au(100) surface. In our studies, the unmodulated structure appears more frequently than the modulated one. The proposed models for both the across and along structures with respect to the clean surface are presented in Figure 3. The PEN ball models, which form each structure, were inserted in the STM image with the atomic resolution of the hex phase of Au(100). The hex stripes are clearly resolved in the STM image; the simplified unit cell as well as the crystallographic and the hex atomic row directions are then assigned. For the modulated structure, drawn in the upper part of the image, two molecules fall on the width of the simplified unit cell of Au(100), and, thus, the PENs are adsorbed, alternating on the ridges and in the valleys. The molecular row was rotated by 18° from the [0$\bar{1}$1] direction, which is consistent with the PEN arrangement visible in Figure 2b,c. For the non-modulated structure, presented in the lower part of the image, one molecule falls on the width of the simplified Au(100) unit cell. It is worth noting that the Au stripe width 14.4 Å [20] is comparable to the length of a PEN molecule 14.2 Å in the gas phase [35].

For both structures, the corresponding distances between neighboring molecules (i.e., side to side or end to end) are comparable. Moreover, the distances are consistent (within measurement accuracy) with those determined by STM studies in the same system in Ref. [14]. Values of 7.4 ± 0.4 Å (side to side) and 15.5 ± 0.8 Å (end to end) were reported for the PEN molecules arranged in molecular rows without modulation in their STM contrasts [14].

**Figure 4:**
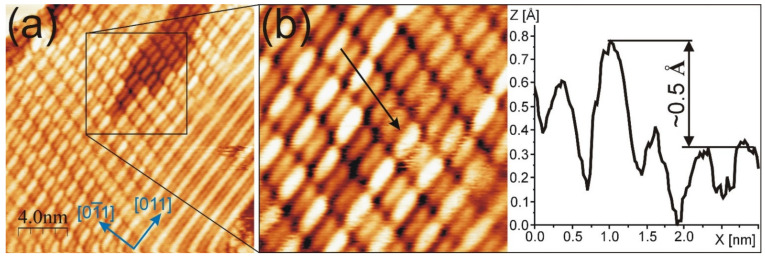
(**a**) STM image of modulated PEN structure, which coexists with PEN domain located on the (1×1) phase of Au(100); (**b**) magnification of (**a**) with the height profile drawn along the black arrow. (**a**,**b**) Taken for U = −0.76 V; I = 0.22 nA.

The coexistence of the modulated PEN structure with a homogenous molecular domain located in the depression was found. It is presented in Figure 4a and its magnification in Figure 4b. The modulated structure surrounds the surface area covered with the non-modulated PEN domain, which is in the upper right quarter of the STM image. The non-modulated domain appears about 0.5 Å lower than the bright molecular feature in the modulated structure, which illustrates the height profile drawn along the green arrow in Figure 4b. This value is close to the height difference between the hex and (1×1) phases (0.6–0.9 Å) in the first layer of the bare and ion-modified Au(100) [22].

In Figure 4, it is clearly visible that molecules adsorbed in the depression have the same adsorption configurations as those on the hex-reconstructed substrate. From two up to six PEN molecules are adsorbed in the depression rows from the lower to the upper part of the image. PEN rows for the modulated structure are aligned with those in the depression. For this arrangement, the rows are rotated by 15 ± 3° from the [0$\bar{1}$1] surface crystallographic direction. The surrounding modulated structure has a patchy pattern, i.e., the pattern significantly differs from place to place. On the right side of Figure 4a, the end-to-end PEN alignment, in which the nearest neighbor molecules have the same apparent height, is observed. Such alignment is not observed in the remaining part of the image. This suggests a change in the underlying template induced by the adsorbate. The change can be compared with that directly observed after the ion modification of the hex-reconstructed Au(100) reported in [22], where stripes with partially non-alternating hex atoms along the same row are presented. The length of such atomic rows is about 50*a*, which is room for around ten PENs adsorbed in the end-to-end manner.

Since the bare Au(100) surface on which PEN was deposited consists of hex-reconstructed structures, the presence of the (1×1) phase indicates that the PEN molecules partially lift the reconstruction. The partial lifting of the Au(100) reconstruction under the influence of 1 ML of α-6T molecules was reported in Ref. [24]. The possibility of hex-Au(100) surface deformation by physisorbed molecules was presented therein. The mechanism of changing the reconstruction was proposed based on the DFT calculations performed for α-6T molecules adsorbed on the hex ridge. The calculations show that the inversion of surface reconstruction by creating the valley with a molecule is more energetically favorable than sliding down α-6T in the nearest valley [24]. To indicate the processes that occur as the Au(100) reconstruction is lifted by PEN, further experimental and theoretical investigations are needed. In contrast to Au(100), the adsorption of 1 ML of PEN or α-6T on Au(111) does not affect the herringbone reconstruction [29,36]. That indicates the influence of surface reconstruction, which is linked with surface electron states [37,38], on the molecule–Au interactions. Our results reveal that the arrangement of PEN on the unreconstructed Au(100) areas corresponds with that occurring in the surrounding domains laying on the hex stripes. Contrary to PEN, α-6T molecules exhibit a different structure in the stripe-like regions (ascribed to the (1×1) phase) than that in the large domains on the reconstructed surface [24]. The arrangement of α-6T on the Au(100)-(1×1) phase was comparable to that observed on the Ag(100) surface, from which no reconstruction proceeds [24]. In the case of PEN on the coinage and unreconstructed metal (100) surfaces, the non-existence of long-range ordered domains was found for 1 ML on Cu(100) at RT [39]. However, the MA was oriented along the high-symmetry axes of the surface [39], which is comparable to the configurations of PEN on the Au(100)-(1×1) phase found in this work.

Our results show that the hex template can be resolved in the STM images, depending on the PEN configuration with respect to the stripes. In the case of non-modulated structures, only the coexistence of phases allows for the identification of a gold structure under the molecular layer. In this work, the (1 × 1) gold phase appears after PEN adsorption. However, it was previously shown that the coexistence of both phases can be obtained for bare Au(100) by ion irradiation [22]. The ion modification of Au(100) surfaces provides the possibility of preparing various templates, which may allow the determination of the substrate structure under a molecular layer.

## 3. Materials and Methods

The measurements were performed under ultra-high vacuum (UHV) conditions at room temperature (RT) in the UHV chamber, hosted with Aarhus 150 STM (SPECS, Berlin, Germany). The STM images were recorded in the constant current mode with a tungsten tip. A negative bias voltage polarity refers to an occupied state imaging (the sample potential). STM images were analyzed with WSxM software, version 5.0 [40], where the following filters were used: equalizing, flattering, inverse fast Fourier transform (FFT), planning, and smoothing. The parameters obtained from the STM studies, e.g., the distance between nearest neighbor molecules, were determined as the average values of at least twenty measurements of a given parameter on various STM images. The experimental errors for those parameters were calculated as the standard deviation.

The Au(100) sample was cleaned by cycles of argon ion sputtering with the ion current, and energy of about 3 μA and 3 keV, followed by annealing at ca. 900 K. After this procedure, the STM images revealed surface terraces with an average width of 400 Å. The cleaning procedure led to the presence of both the rotated and non-rotated hex-reconstructed topmost layer of Au(100). The (1×1) phase was not observed.

PEN molecules (from SigmaAldrich,(Saint Louis, USA) purity 99%) were deposited from a home-made effusion cell. One monolayer herein refers to the amount of flat-deposited PEN molecules that completely cover the substrate surface. The deposition rate was about 0.1 ML/min.

## 4. Conclusions

Utilizing the STM, we studied the structural properties of 1 ML of PEN deposited on the hex Au(100) surface at RT. Two molecular structures on the reconstructed substrate were found. The PEN arrangements differ by the orientation of MA with respect to hex stripes. For PENs with their MA laid across the hex-atomic rows, the molecular structure is non-modulated. The molecular rows are oriented along the hex stripes, and a single PEN is adsorbed across the width of a simplified Au(100) unit cell. The long-range modulation in the apparent height of PENs, associated with molecule adsorption on the ridges and in the valleys of hex stripes, is observed in the structure where MA is along the atomic rows. Two molecules fall on the width of a simplified Au(100) unit cell. The direction of molecular rows with respect to the <011> direction may rotate up to 25°. The side-to-side and end-to-end distances are the same within the experimental errors for both arrangements.

The coexistence of PEN domains on hex Au(100) with those on the (1×1) phase was found for both structures. This indicates that 1 ML of PENs partially lifts the substrate reconstruction. The configuration of PENs as well as the orientation of molecular rows on Au(100)-(1×1) are in agreement with those of the surrounding domains on the hex gold structure. The apparent height difference between the hex and (1×1) phases of Au(100) is preserved under the PENs layer and in the range of tenths of angstrom. For the modulated structure, the alignment of PENs with a homogenous STM contrast along the stripes is observed, which is the sign of the rearrangement of atoms in the underling hex surface induced by an adsorbate.

## Data Availability

The raw data will be made available upon request.

## Sample Availability

Samples are available commercially.
